# Impact of treatment for opioid dependence on fatal drug‐related poisoning: a national cohort study in England

**DOI:** 10.1111/add.13193

**Published:** 2015-11-25

**Authors:** Matthias Pierce, Sheila M. Bird, Matthew Hickman, John Marsden, Graham Dunn, Andrew Jones, Tim Millar

**Affiliations:** ^1^ Institute of Brain Behaviour and Mental Health, Faculty of Medical and Human SciencesUniversity of ManchesterUK; ^2^ Institute of Population Health, Faculty of Medical and Human SciencesUniversity of ManchesterUK; ^3^ MRC Biostatistics UnitCambridgeUK; ^4^ School of Social and Community MedicineUniversity of BristolUK; ^5^ Addictions Department, Institute of Psychiatry, Psychology and NeuroscienceKing's College LondonUK

**Keywords:** Drug‐related poisoning, opiate dependence, opioid agonist pharmacotherapy, overdose, psychosocial treatment, residential treatment

## Abstract

**Aims:**

To compare the change in illicit opioid users’ risk of fatal drug‐related poisoning (DRP) associated with opioid agonist pharmacotherapy (OAP) and psychological support, and investigate the modifying effect of patient characteristics, criminal justice system (CJS) referral and treatment completion.

**Design:**

National data linkage cohort study of the English National Drug Treatment Monitoring System and the Office for National Statistics national mortality database. Data were analysed using survival methods.

**Setting:**

All services in England that provide publicly funded, structured treatment for illicit opioid users.

**Participants:**

Adults treated for opioid dependence during April 2005 to March 2009: 151 983 individuals; 69% male; median age 32.6 with 442 950 person‐years of observation.

**Measurements:**

The outcome was fatal DRP occurring during periods in or out of treatment, with adjustment for age, gender, substances used, injecting status and CJS referral.

**Findings:**

There were 1499 DRP deaths [3.4 per 1000 person‐years, 95% confidence interval (CI) = 3.2–3.6]. DRP risk increased while patients were not enrolled in any treatment [adjusted hazard ratio (aHR) = 1.73, 95% CI = 1.55–1.92]. Risk when enrolled only in a psychological intervention was double that during OAP (aHR = 2.07, 95% CI = 1.75–2.46). The increased risk when out of treatment was greater for men (aHR = 1.88, 95% CI = 1.67–2.12), illicit drug injectors (aHR = 2.27, 95% CI = 1.97–2.62) and those reporting problematic alcohol use (aHR = 2.37, 95% CI = 1.90–2.98).

**Conclusions:**

Patients who received only psychological support for opioid dependence in England appear to be at greater risk of fatal opioid poisoning than those who received opioid agonist pharmacotherapy.

## Introduction

Non‐medical use of opioid drugs is associated with a significant global burden of disease 1. In the United Kingdom, 1% of the illicit opioid‐using population dies each year 2, 3. More than half of these deaths are due to respiratory failure following accidental overdose 3, 4, 5.

Opioid agonist pharmacotherapy (OAP) is a community treatment for opioid dependence which aims to reduce heroin and other non‐medical opioid use and associated harm. Using oral methadone or buprenorphine, well‐delivered OAP manages the patient's physiological dependence, attenuates drug use cravings and facilitates access to health‐care and recovery supports. Meta‐analyses of randomized controlled trials show that OAP is effective at retaining patients in treatment and reducing heroin use 6, 7, 8. The World Health Organization (WHO) and the National Institute for Health and Care Excellence (NICE) recommend OAP as the front‐line maintenance treatment for opioid dependence 9, 10. Oral methadone and buprenorphine are also used for medically supervised withdrawal in community and hospital settings.

Most developed health‐care systems also provide psychological support interventions to treat opioid dependence. Guided by individual need, preference and any previous clinical response, these interventions are offered either concurrently or sequentially with OAP, or they are offered as a stand‐alone treatment with no substitute medication.

Widely delivered in the United Kingdom, community‐setting psychological support comprises a broad set of structured change methods which aim to reduce the opioid‐dependent person's cognitive behavioural symptoms, intra/interpersonal difficulties and social problems and build motivation for recovery. The patient is assigned a clinical key worker to help develop, implement and review their care plan 11. Psychological support interventions are also provided by residential rehabilitation services; these programmes are guided by a characteristic philosophical approach and vary in duration and intensity.

NICE recommends that people with drug‐related problems are provided with information about self‐help groups and that those outside structured treatment are offered brief motivational interventions 12. NICE endorses contingency management (which uses practical reinforcers to motivate adherence to treatment and behaviour change) as an adjunctive therapy during OAP but judges that there is insufficient evidence to recommend routine use of cognitive behavioural therapy or psychodynamic therapy. Standalone psychological support for opioid dependence is also not recommended—with the exception of behavioural couples therapy for those who have an appropriate non‐drug‐misusing partner.

Observational studies of addiction treatment systems have reported that the risk of fatal drug‐related poisoning (DRP) is at least halved when patients are enrolled in treatment for opioid dependence 5, 13, with this risk increasing immediately following the start of treatment and after it ends 14, 15. However, to date, estimation of the change in DRP risk associated with psychological support interventions has been hampered by limited statistical power in small‐scale controlled trials and meta‐analyses 16.

Large‐scale research is therefore needed to inform policy and service providers about the DRP risk associated with psychological support interventions and identify modifiers of treatment impact. Isolating the individual effect of a particular treatment is challenging, because people may receive several different treatments in an episode of care and over time 17. An episode of treatment can comprise a single intervention, or several interventions delivered in combination or sequence (e.g. OAP then psychological intervention; psychological support followed by residential rehabilitation; and so on).

Previous studies have identified male gender, older age, illicit drug injecting and concurrent use of central nervous system depressants (e.g. alcohol and benzodiazepines) as independent risk factors for DRP 3, 5, 14, 18. We included these as potential modifiers of treatment effects along with two additional measures: referral from the criminal justice system (CJS); and whether the clinical service reported that the patient had completed their treatment successfully.

Approximately a quarter of treatment admissions in England are CJS referred 19, and this subpopulation is less likely to abstain from heroin during treatment 20. Most CJS‐referred treatment involves standard provision of care, with a very small minority of cases mandated to treatment by the court. The goal of completing treatment abstinent from opioid use (illicit or otherwise) has been a recent priority for a recovery‐orientated policy in the United Kingdom 21, 22.

To investigate the DRP risk for community psychological support and residential interventions and compare it with the risk associated with OAP, we developed a national Drug Data Warehouse 23 project to link treatment and mortality data in England. We asked three questions in this study:

1. What is the DRP risk associated with time patients spend in treatment and time spent out of treatment?
2. What is the DRP risk associated with psychological support in comparison to OAP?
3. Is the association between treatment and DRP risk moderated by: referral and patient characteristics; the first month after admission and discharge; and by successful completion of treatment?

## Methods

### Design

This was a national data linkage study of the English National Drug Treatment Monitoring System (NDTMS) and the Office for National Statistics (ONS) mortality database. NDTMS is a national system that monitors the delivery of all public treatment for psychoactive substance‐related problems by National Health Service and third‐sector providers 24, which together account for almost all such provision in England. The ONS mortality database includes all registered deaths in England and Wales. Data extracted from these databases were linked for a 4‐year observation period (1 April 2005 to 31 March 2009).

### Patient and treatment information

NDTMS records four types of treatment: community OAP, community psychological support, in‐patient withdrawal management and drug‐free residential rehabilitation. Enrolment in in‐patient and residential treatment each provided a small number of person‐years (1224 and 2601, respectively). We noted that this reduced statistical power for an intervention level analysis. As both interventions are abstinence orientated, we judged it appropriate to create a combined grouping (labelled ‘residential’).

**Figure 1:**
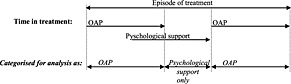
Schematic diagram representing the categorization of treatment modalities

During the observation period, 191 310 adults (aged 18–64 years) were treated for opioid dependence (for 1 or more days). Some patients received one treatment episode, while others received several episodes during this time. The date on which each patient started and ended each treatment enabled us to classify time spent in the following mutually exclusive groups: residential (with or without OAP or psychological support); OAP (with or without psychological support); and psychological support alone (Fig. 1 shows a schematic illustration of the construction of these treatment groups).

For each opioid treatment episode, the following clinic admission information from NDTMS was used for the analysis: gender; age; referral source; self‐reported illicit drug injecting status (past month); and an optional self‐report of up to three additional problematic psychoactive substances which were relevant at admission 25. The status of the patient at discharge was categorized according to the assessment of the clinical service as either ‘successfully completed’ or ‘not completed’. Only illicit drug injecting status included a ‘not answered’ code for missing data.

### DRP mortality

The ONS provided data on all deaths occurring during the observation period which were registered by 30 September 2011. This allowed for delays in the registration process pending inquest verdicts, as recommended for research 26.

Deaths due to DRP were identified from the following WHO International Classification of Disease (ICD‐10) codes: ‘mental and behavioural disorders due to psychoactive substance use, excluding alcohol and tobacco’ (F11–16, F18–19); ‘accidental poisoning by drugs, medicaments and biological substances’ (X40–X44); ‘intentional self‐poisoning drugs, medicaments and biological substances’ (X60–X64); ‘assault by drugs, medicaments and biological substances’ (X85); and ‘poisoning by drugs, medicaments and biological substances, undetermined intent’ (Y10–Y14) 27.

### Data linkage procedure

Treatment and mortality data were linked using a minimal identifier in each database (initials, date of birth, gender) and government region of usual residence. During preparatory work, we noted that when linking NDTMS data with person‐unique CJS identifiers, up to 22% of minimal identifiers in the treatment population were shared by one or more individuals. This is an overestimation of the true rate at which patients shared the same identifying characteristics because CJS identifiers are entered manually into the database and are subject to error. Conservatively, all such records were excluded from the analysis to reduce false positive matching. This created a cohort of 151 982 individuals for the present study. All patient identifiers were irreversibly encrypted to ensure that features of the original data could not be discerned.

Following advice on the study procedure from the NHS Central Office for Research Ethics Committees and the office of the University of Manchester Research Ethics Committee, approval for access to NDTMS and mortality records was secured from the English National Treatment Agency for Substance Misuse and the ONS Microdata Release Panel, respectively.

### Statistical analysis

Unadjusted and adjusted proportional hazard ratios (HR and aHR) for the DRP risk were calculated by Cox regression (Stata release 13) with the calendar–time origin for survival set to 1 April 2005.

In‐treatment status was defined according to the dates of admission/discharge for each treatment episode, identified by time‐dependent variables. Time spent out of treatment started from the day after the end of a treatment episode to the day prior to the start of a new episode (or to end of follow‐up if there were no further interventions recorded). Individuals in the cohort entered the DRP risk‐set on 1 April 2005 if they were already enrolled in treatment, or they entered the risk‐set from the start of their first treatment episode during the observation period. Cohort members ceased to be at risk at 31 March 2009, or from the date of their death from any cause.

Typically, patients in the UK treatment system are expected to attend their clinical service fortnightly or more often. We noted that the discharge date could indicate erroneously that a DRP occurred out‐of‐treatment because the clinical service recorded the patient's last face‐to‐face clinical contact as their discharge date (as per NDTMS guidance 25). We observed 130 deaths associated with OAP and 18 associated with psychological support within 2 weeks of discharge where the discharge reason was ‘died’. These were assigned as in‐treatment deaths by extending the date of discharge.

We adjusted the analysis for the following covariates judged influential for DRP risk 2, 3 or potential confounders of DRP risk and treatment 20: gender; time‐updated age (categorized as 18–35, 35–44, 45–64 years); patient‐reported injecting status; patient‐reported problematic use of alcohol, benzodiazepines, crack cocaine (the smokeable base form), cocaine powder and amphetamines (the latter two substances combined as ‘other stimulants’ because of a low level of reporting); and referred from CJS (analysis focus).

The following time‐dependent covariates were constructed:

1. the risk‐set for illicit drug injecting and additional problematic substances was defined from the date of first report (as this occurred). Persons remained in that risk‐set until the end of the observation period;
2. the risk‐set for CJS referral was defined from the date of treatment admission and this was updated (as necessary) for a subsequent episode; and
3. discharge status (completed or not completed) was defined for the periods following discharge, until the beginning of the next treatment‐episode or the end of the observation period.

We fitted the following pre‐specified interactions to investigate effect modification for the relationship between treatment and changed DRP risk, with statistical significance set at *P* < 0.01: gender; age (over 35 or not); DRP behavioural risks (injecting, problematic alcohol and benzodiazepine use); and CJS referral. Effect‐modification was assessed by individually fitting each of these six interactions and testing their statistical significance by likelihood ratio tests. To test joint effects of each reported interaction, all six interactions were included in a single, adjusted model.

The following time‐dependent indicators were used to investigate DRP risk following admission and discharge: the 28 days following admission; the intervening time enrolled in each treatment; and the 28 days following discharge (providing no further treatment was received). If an episode comprised a sequence of more than one type of treatment (defined hierarchically), the first 28 days out of treatment was assigned to discharge from the last treatment. The referent category was the intervening period enrolled in OAP. Using a categorical time‐dependent variable, the out‐of‐treatment DRP risk for those classified as having completed treatment successfully was compared with the risk for those who did not successfully complete treatment. Graphical plots of Schoenfeld residuals were used to check for the proportional hazard assumption in all regression models.

There were two sensitivity checks. First, given the likelihood of imprecision in the reporting of treatment episode dates, the analyses were repeated with discharge dates extended by 14 days for patients who did not complete treatment. Secondly, to allow for length‐biased accrual, analyses were performed separately for treatment episodes which started before and those which started on or after 1 April 2005.

## Results

Between 1 April 2005 and 31 March 2009, the 151 983 individuals in the cohort contributed 4423.4 person‐years (PY) of observation and 236 660 treatment episodes to the analysis (Table 1).

**Table 1:** Cohort description, follow‐up time, deaths and covariates (*n*=151 983 individuals).

*Category*	*Full sample*
Demographics	69%
Total number of individuals	151 983
Number (%) of individuals who were	
Male	105 172 (69)
Female	46 811 (31)
Median age (IQR) at cohort entry, years	32.6 (27.2, 38.9)
Observation period	
Total person‐years of observation	442 950
Number (%) of person‐years of observation spent	
Out of any treatment	135 864 (31)
In any treatment	307 086 (69)
Number (%) of person‐years in treatment according to type of intervention received	
Pharmacotherapy	272 280 (89)
Psychological support	30 977 (10)
Residential	3825 (1)
Median person‐years (IQR) of observation per individual	3.4 (2.0, 4.0)
Treatments	
Total number of treatment episodes	236 660
Number (%) of treatment episodes that included each intervention type^a^	
Pharmacotherapy	190 339 (80)
Psychological support	69 033 (29)
Residential	17 193 (7)
Number (%) of individuals who received (during observation period)	
One treatment episode	99 416 (65)
Two treatment episodes	32 285 (21)
Three treatment episodes	12 711 (8)
Four treatment episodes	4813 (3)
Five or more treatment episodes	2758 (2)
Deaths	
Total number recorded during observation period	3503
Number (%) of deaths that were due to	
Drug‐related poisoning	1499 (43)
Other causes	2004 (57)

a Percentages here round to > 100% because an episode could include more than one type of intervention. IQR = interquartile range.

During the study there were 1499 DRP‐related deaths: a DRP mortality rate of 3.4 per 1000 PY [95% confidence interval (CI) = 3.2–3.6]. The following covariates were associated with an increase in DRP risk: increasing age, drug injecting, problematic alcohol use, problematic benzodiazepine use and male gender (Table 2). Covariate associations were stable across all regression models (see Appendix A in Supporting information).

**Table 2:** Unadjusted and covariate adjusted Cox regression of risk of fatal drug‐related poisoning (DRP) during time in and out of treatment (*n* = 151 983 individuals).

*Variable*	*Person‐years, 1000*	*No. of DRPs*	*DRP rate, per 1000 person‐years*	*HR*	*aHR*	*P‐value*
Status						
In treatment	307	890	2.9 (2.7, 3.1)	1	1	< 0.001
Not in treatment	136	609	4.5 (4.1, 4.9)	1.57 (1.42, 1.75)	1.73 (1.55, 1.92)	
Covariates						
Gender						
Male	303	1162	3.8 (3.6, 4.1)	1	1	< 0.001
Female	140	337	2.4 (2.2, 2.7)	0.63 (0.56, 0.71)	0.70 (0.62, 0.79)	
Age group (years)						
18–34	236	608	2.6 (2.4, 2.8)	0.63 (0.57, 0.71)	0.64 (0.57, 0.72)	< 0.001
35–44	150	608	4.1 (3.7, 4.4)	1	1	
45–64	57	283	5.0 (4.4, 5.6)	1.23 (1.07, 1.41)	1.31 (1.13, 1.51)	
Drug injecting^a^						
Yes	163	788	4.8 (4.5, 5.2)	2.15 (1.93, 2.41)	2.12 (1.89, 2.37)	< 0.001
No	235	526	2.2 (2.1, 2.4)	1	1	
Not declared	45	185	4.1 (3.6, 4.7)	1.82 (1.54, 2.16)	1.77 (1.50, 2.10)	
Alcohol^a^						
Yes	55	313	5.6 (5.1, 6.3)	1.84 (1.64, 2.10)	1.72 (1.52, 1.95)	< 0.001
No	387	1186	3.1 (2.9, 3.2)	1	1	
Benzodiazepines^a^						
Yes	60	288	4.8 (4.3, 5.4)	1.53 (1.34, 1.74)	1.44 (1.26, 1.64)	< 0.001
No	383	1211	3.2 (3.0, 3.3)	1	1	
Crack cocaine^a^						
Yes	162	559	3.4 (3.2, 3.7)	1.03 (0.93, 1.15)	0.98 (0.88, 1.09)	0.71
No	280	940	3.4 (3.1, 3.6)	1	1	
Other stimulants^a^						
Yes	58	227	3.9 (3.4, 4.5)	1.19 (1.03, 1.37)	1.10 (0.96, 1.27)	0.17
No	385	1272	3.3 (3.1, 3.5)	1	1	
Following CJS referral^b^						
Yes	82	279	3.4 (3.0, 3.8)	1.01 (0.89, 1.16)	0.97 (0.85, 1.11)	0.65
No	361	1220	3.4 (3.2, 3.6)	1	1	

Numbers in parentheses are 95% confidence intervals (CIs); DRP, fatal (opioid) drug‐related poisoning; HR = hazard ratio; aHR = adjusted HR (for all other variables); HR/aHR = value of 1 denotes baseline category.

a Patient reported: additional, concurrent problem to opioid dependence at treatment assessment (time dependent covariate; not declared are missing data).

b From start to subsequent treatment‐episode. CJS = criminal justice system.

### Risk in and out of treatment

During treatment the DRP mortality rate was 2.9 (95% CI = 2.7–3.1) per 1000 PY and was 4.5 (95% CI = 4.1–4.9) per 1000 PY during periods out of treatment (Table 2). After adjustment, DRP risk was associated strongly with periods spent out of any treatment.

Table 3 shows the risk modification for treatment enrolment status by referral and patient characteristics. The overall association of treatment with reduced DRP risk was substantially greater for males (*P* = 0.002), illicit drug injectors (*P* < 0.001), and patients who reported problematic alcohol use (*P* = 0.002). There was insufficient evidence of a treatment effect for patients referred from the CJS in contrast to non‐CJS referred patients (*P* < 0.001).

**Table 3:** Cox regression of DRP risk during time in and out of treatment modified by referral and patient characteristics (*n =* 151 983 individuals).

*Effect modifier*	*Treatment status*	*Person‐years, 1000*	*No. of DRPs*	*DRP rate, per 1000 PY*		*HR*	*aHR*	*P‐value* a
Gender								
Male	In	207	659	3.2	(2.9, 3.4)	1	1	0.002
	Out	96	503	5.2	(4.8, 5.7)	1.68 (1.49, 1.88)	1.88 (1.67, 2.12)	
Female	In	100	231	2.3	(2.0, 2.6)	1	1	
	Out	40	106	2.7	(2.2, 3.2)	1.17 (0.93, 1.47)	1.26 (1.00, 1.59)	
Injecting								
Injector	In	121	457	3.8	(3.4, 4.1)	1	1	< 0.001
	Out	42	331	7.9	(7.1, 8.8)	2.13 (1.85, 2.46)	2.27 (1.97, 2.62)	
Not injector/undeclared	In	186	433	2.3	(2.1, 2.6)	1	1	
	Out	94	278	3.0	(2.6, 3.3)	1.30 (1.11, 1.51)	1.36 (1.17, 1.58)	
Alcohol								
Yes	In	40	172	4.3	(3.7, 5.0)	1	1	0.002
	Out	16	141	9.1	(7.7, 10.7)	2.16 (1.73, 2.70)	2.37 (1.90, 2.98)	
No	In	267	718	2.7	(2.5, 2.9)	1	1	
	Out	120	468	3.9	(3.6, 4.3)	1.48 (1.31, 1.66)	1.59 (1.41, 1.79)	
CJS referral								
Yes	In	48	164	3.4	(2.9, 4.0)	1	1	< 0.001
	Out	34	115	3.4	(2.8, 4.1)	1.01 (0.80, 1.28)	1.15 (0.90, 1.46)	
No	In	259	726	2.8	(2.6, 3.0)	1	1	
	Out	102	494	4.8	(4.4, 5.3)	1.76 (1.56, 1.97)	1.90 (1.69, 2.14)	

Numbers in parentheses are 95% confidence intervals (CIs); HR = hazard ratio; DRP = fatal (opioid) drug‐related poisoning; aHR = adjusted HR (for all variables present in Table 2—see Appendix A for covariate estimates); HR/aHR = value of 1 denotes baseline category.

a Likelihood ratio tests of comparison with adjusted model in Table 2 (i.e. test for effect modification). Two further variables tested for effect modification, but with *P* > 0.01 (adjusted models): benzodiazepine use *P =*  0.03; age *P =* 0.87. All interactions were fitted individually in adjusted model. As a sensitivity check, all interactions were included in a single, adjusted model to test their independence: the results were very similar to those reported here. DRP = drug‐related poisoning; CJS = criminal justice system.

### Risk and treatment type

After covariate adjustment, the DRP risk associated with community psychological support was twice that associated with OAP (Table 4). There was no evident difference in risk between periods spent in residential treatment and periods in OAP.

**Table 4:** Cox regression of DRP risk by discharge status, type of treatment, and time spent in and out of treatment (*n*=151 983 individuals).

*Model*	*Characteristic*	*Person‐years, per 1000*	*N of DRPs*	*DRP rate, per 1000 person‐years*		*HR*		*aHR*		*P‐value* a
Completed treatment	In treatment	307	890	2.9	(2.7, 3.1)	1		1		0.11
	Out of treatment									
	Completed successfully	37	145	3.9	(3.3, 4.6)	1.37	(1.14, 1.63)	1.54	(1.29, 1.84)	
	Not Completed Successfully	98	464	4.7	(4.3, 5.2)	1.65	(1.47, 1.85)	1.79	(1.59, 2.01)	
Intervention	In treatment, modality received									< 0.001
	Residential	3.8	15	3.9	(2.4, 6.5)	1.50	(0.90, 2.49)	1.28	(0.76, 2.13)	
	OAP	272	712	2.6	(2.4, 2.8)	1		1		
	Psychological support	31	163	5.3	(4.5, 6.1)	2.00	(1.69, 2.38)	2.07	(1.75– 2.46)	
	Out of treatment	136	609	4.5	(4.1, 4.9)	1.74	(1.56, 1.94)	1.92	(1.72, 2.15)	
Periods in/out of treatment intervention	Residential									< 0.001
	In treatment^b^	3.8	15	3.9	(2.4, 6.5)	1.48	(0.89, 2.48)	1.20	(0.72, 2.01)	
	1–28 days following discharge^c^	0.5	10	18.8	(10.1, 35.0)	7.01	(3.72, 13.23)	6.06	(3.21, 11.46)	
	OAP									
	1–28 days following admission	22	42	1.9	(1.4, 2.6)	0.69	(0.50, 0.97)	0.58	(0.41, 0.81)	
	Remainder in treatment	250	670	2.7	(2.5, 2.9)	1		1		
	1–28 days following discharge^c^	7	63	9.3	(7.3, 11.9)	3.56	(2.74, 4.62)	3.59	(2.77, 4.67)	
	Psychological support									
	1–28 days following admission	7	31	4.7	(3.3, 6.7)	1.69	(1.14, 2.51)	1.44	(0.97, 2.15)	
	Remainder in treatment	24	132	5.4	(4.6, 6.4)	2.09	(1.73, 2.53)	2.08	(1.72, 2.52)	
	1–28 days following discharge^c^	3	11	3.9	(2.2, 7.0)	1.53	(0.84, 2.78)	1.52	(0.83, 2.77)	
	Over 28 days out of any treatment	126	525	4.2	(3.8, 4.5)	1.58	(1.41, 1.78)	1.68	(1.50, 1.90)	

Numbers in parentheses are 95% confidence intervals (CIs); HR = hazard ratio; DRP = fatal (opioid) drug‐related poisoning; OAP = opioid agonist pharmacotherapy aHR = adjusted HR (for all variables present in Table 2—see Appendix A for covariate estimates); HR/aHR value of 1 denotes the baseline category.

a Likelihood ratio tests of comparison with adjusted model in Table 2.

b Merged with 1–28 days in residential treatment care, as no DRP deaths in this time period (883 person‐years).

c Discharged from treatment and excluding any periods where client was transferred to another treatment.

### DRP risk early in treatment and after discharge

The first 28 days of OAP were associated with a lower DRP risk than that associated with the period enrolled in this treatment thereafter (Table 4). There was no evidence of changed DRP risk between the first month and the remainder of psychological support. There were no deaths in the first 28 days after admission to residential treatment.

The DRP risk increased during the month immediately following discharge from OAP but there was weaker evidence of an increase during the month following discharge from psychological support. Patients discharged from residential treatment had approximately twice the risk associated with discharge from OAP. There was evidence that the increased DRP risk persisted for the period beyond the first month following discharge from any treatment.

There was insufficient evidence for a difference in post‐treatment DRP risk between patients judged to have completed treatment successfully and those who did not (*P* = 0.11).

### Sensitivity analyses and assumption checks

We extended the recorded date of discharge for non‐completed treatment by 14 days and also repeated the analyses separately for episodes which started before or after 1 April 2005 to assess duration‐biased accrual. These analyses did not influence the inferences made (see Appendices B and C, Supporting information). Following each model fitted, graphical analysis of the Schonfeld residuals revealed no substantial departures from the vassumption of proportional hazards.

## Discussion

We observed an elevated DRP risk during periods out of any treatment for opioid dependence. During treatment there was a greater reduction in this risk for men, for illicit drug injectors and those who reported problematic alcohol use and, consistent with meta‐analysis 6, 7, 8, OAP was associated with a strong reduction in DRP risk. The DRP risk increased during the month following discharge from OAP or residential treatment and elevated risk persisted beyond the month following discharge.

The DRP risk associated with psychological support was twice that for OAP (aHR = 2.07, 95% CI = 1.75–2.46) and was comparable to the risk when not in treatment. This is consistent with an earlier observation that ‘drug‐free’ treatment is associated with a higher all‐cause mortality risk 28. This is unlikely to reflect elevated risk on transition from OAP to psychological support. In fact, this clinical pathway was extremely rare in the cohort and this transition occurred in only 1% of treatment episodes (see Appendix D, Supporting information).

There was no evidence that completing treatment successfully was associated with a reduction in DRP risk, nor was DRP risk reduced during treatment among people referred from the CJS. We conducted a *post‐hoc* analysis to determine whether this might be due to the inclusion of those referred following release from prison, because this confers a substantially elevated risk of mortality 29. However, the association between treatment and risk remained weak, even for non‐prison CJS referrals (see Appendix E, Supporting information).

In contrast to previous studies, we found no elevation in risk at OAP treatment onset. This may reflect more effective recent adherence to guidelines for initiation of opioid prescribing at the predominantly specialist treatment settings studied here compared to the primary care setting of an earlier English study 15.

Small‐scale, uncontrolled studies have observed reductions in drug use among CJS referrals 30, 31. However, the present results concur with findings from a large‐scale cohort indicating that CJS referral is associated with a reduced likelihood of the patient achieving abstinence or reducing drug use 20. It has been suggested that the crime reduction and administrative demands in the CJS may limit time for clinical interventions to treat dependence 32.

Our study has several strengths. This is the largest cohort study on DRP risk to be published to date, comprising England‐wide data from all publicly funded opioid dependence treatment services in the NHS and third‐sector providers. It afforded sufficient statistical power to explore interactions and comparison of DRP risk for OAP and psychological support interventions. The findings were also robust to sensitivity analysis around the date of treatment discharge and between prevalent and incident treatment episodes (Appendices B and C, respectively; Supporting information). The data linkage design delivered minimal loss to follow‐up, although we note that this design is not able to account for cohort loss due to people who leave the country.

We also acknowledge several limitations. First, the start date for OAP is more clearly identified than its end date, because treatment cessation may only become apparent once a patient has failed to present for a repeat prescription. Our approach to recording end‐of‐treatment differed from those in some previous studies (e.g. utilizing prescription end‐date) 15. Prescription data may have provided better information on when medication was provided. However, prescriptions may continue to be issued beyond when patients cease to collect them. In Australia, Degenhardt and colleagues defined a period after the end of recorded discharge when the patient was assumed still to be in treatment 14, whereas our approach assumed this only if we had a prior expectation (i.e. when discharge was recorded as being due to death; *n* = 148).

Secondly, the observational design limits the capacity to make causal inferences. Uncontrolled confounding may account for some of the differences in DRP risk reported, and we lacked case‐mix information on opioid dependence severity and co‐existing health and social problems to strengthen the analysis. Also, because the variables describing behavioural risk factors are interval‐censored (i.e. information is only available at the start of treatment), controlling for these is likely to account for only part of the confounding. However, adjustment for the evidence‐supported covariates that were available indicated confounding away from the null (unadjusted HR = 2.00; aHR = 2.07).

Thirdly, NDTMS data did not specify the specific methods of psychological support received by patients; thus, variability in the receipt of such treatment could not be explored. Similarly, it was not possible to distinguish the relative effects of methadone and buprenorphine within OAP, and this may have led to a loss of information. Both of these are accessible in the publicly funded, community‐based UK treatment setting. We note recent work indicating a lower risk associated with buprenorphine during early treatment 33, although previous UK research has not differentiated the DRP risk by agonist medication either early in treatment or post‐discharge 15. Our group is now investigating this issue using another treatment database.

The elevated risks observed on discharge from OAP and residential treatments are consistent with previous studies 14, 15. This is likely to be mediated by reduced opioid tolerance 34, due to dose tapering and cessation of prescribing at discharge. The nature of residential treatment may vary across countries; as provided in the United Kingdom it is an intensive, non‐mandatory intervention received by patients who appear more amenable to treatment 35. Further, appropriately powered research is needed to contrast the DRP associated with discharge from in‐patient and residential rehabilitation services.

The treatment classification of successful completion (i.e. a conclusion of the patient's care plan and discharge mutually agreed) has face validity as an indicator of clinical effectiveness. Recent treatment policy in the United Kingdom has emphasized monitoring of this criterion as an important indicator of effectiveness 21, 22. However, this is a proxy indicator of clinical response and service providers may or may not use objective biological testing to document abstinence. We could find little evidence of a difference in DRP risk between those judged to have completed or not completed treatment. It is possible that the former patients faced greater risk of DRP on relapse, while non‐completers had higher levels of opioid tolerance from which they derived some protection 36.

In conclusion, our findings show that: (1) DRP risk is lower during treatment and substantially higher out of treatment; (2) psychological support is associated with twice the risk observed for OAP; and (3) that successfully completing treatment is not associated with a reduction in risk. Because psychological support was the second most common pattern observed in the present study, we recommend that there should be a clear focus upon identifying and reducing overdose risk in patients who receive stand‐alone psychological support for opioid dependence. Opioid overdose should be an explicit discussion topic with patients who present for psychological support.

## Declaration of interests

T.M. has received research funding from the UK National Treatment Agency for Substance Misuse (now Public Health England) and the Home Office. He is a member of the organizing committee for, and chairs, conferences supported by unrestricted educational grants from Reckitt Benckiser, Lundbeck, Martindale Pharma and Britannia Pharmaceuticals Ltd, for which he receives no personal remuneration. S.M.B. holds GSK shares. She chaired Home Office's Surveys, Design and Statistics Subcommittee (SDSSC) when its report on 21st Century Drugs and Statistical Science was published. She has previously served as UK representative on the Scientific Committee for European Monitoring Centre for Drugs and Drug Addiction. She is co‐principal investigator for MRC‐funded, prison‐based N‐ALIVE pilot Trial. J.M. works in an integrated university [Institute of Psychiatry, Psychology and Neuroscience (IOPPN), King's College London (KCL)] and National Health Service Academic Health Sciences Centre (King's Health Partners) and is supported by university‐based research grants from the Department of Health, Institute for Health Research (Health Technology Assessment programme), and the National Institute for Health Research (NIHR) Biomedical Research Centre for Mental Health at South London and Maudsley NHS Foundation Trust (SLaM) and IoPPN/KCL. He declares part‐time employment as Senior Academic Adviser for the Alcohol, Drugs and Tobacco Division, Health and Wellbeing Directorate, Public Health England. He acknowledges the following pharmaceutical industry support: educational grant funding to KCL and SLaM from Indivior PLC via Action on Addiction for a study psychological interventions in opioid dependence (2010‐2016); consultation to Merck Serono (2013, 2015), and honoraria as co‐chair of the Improving Outcomes in Treatment of Opioid Dependence conference (2015) via educational grant funding from Indivior to PCM Scientific. He holds no stocks in any company. A.J. has received research funding from the UK National Treatment Agency for Substance Misuse (now Public Health England) and the Home Office.

## Supporting information




**Appendix S1** Covariates estimates for models in analysis.
**Appendix S2** Sensitivity analysis comparing periods in treatment with periods out after varying the treatment end date to two weeks later than the recorded date for those with an ‘unplanned’ discharge.
**Appendix S3** Sensitivity analysis: Separate proportional hazard analysis for those in treatment 1st April 2005 (prevalent cohort) and those who entered treatment between 1st April 2005 to 31st March 2009 (incident cohort).
**Appendix S4** Most common sequences of treatment interventions within a treatment‐episode, first incident episodes only (number of subjects = 114,741; person years = 196,129).
**Appendix S5** Post hoc analysis to assess the independent impact of CJS referrals from prison and the community.

Supporting info item
