# Supplementary material for: Impact of treatment for opioid dependence on fatal drug‐related poisoning: a national cohort study in England
Source: Addiction. 2015 Nov 25;111(2):298–308. doi: 10.1111/add.13193 (PMC4950033; doi:10.1111/add.13193)
Supplement: Supplementary file 1 — Supporting info item [file ADD-111-298-s001.docx]

**Appendices: Impact of treatment for opioid dependence on fatal drug-related poisoning: a national cohort study in England**

**Appendix A: Covariates estimates for models in analysis**

| **Variable** | **Injecting effect modification model** | | **Alcohol effect modification model** | | **CJS effect modification model** | | **Gender effect modification model** | |
| --- | --- | --- | --- | --- | --- | --- | --- | --- |
| **Gender** |  |  |  |  |  |  |  |  |
| **Male** | 1 |  | 1 |  | 1 |  | - | - |
| **Female** | 0.70 [0.62, 0.79] | <0.001 | 0.70 [0.62, 0.79] | <0.001 | 0.70 [0.62, 0.79] | <0.001 | - |  |
| **Age group** |  |  |  |  |  |  |  |  |
| **18-34** | 0.64 [0.57, 0.72] | <0.001 | 0.64 [0.57, 0.72] | <0.001 | 0.64 [0.57, 0.72] | <0.001 | 0.64 [0.57, 0.72] | <0.001 |
| **35-44** | 1 |  | 1 |  | 1 |  | 1 |  |
| **45-64** | 1.31 [1.13, 1.51] | <0.001 | 1.30 [1.13, 1.50] | <0.001 | 1.31 [1.14, 1.51] | <0.001 | 1.31 [1.13, 1.51] | <0.001 |
| **Injecting *** |  |  |  |  |  |  |  |  |
| **Yes** | - | - | 2.13 [1.90, 2.38] | <0.001 | 2.11 [1.89, 2.36] | <0.001 | 2.12 [1.90, 2.38] | <0.001 |
| **No** | - |  | 1 |  | 1 |  | 1 |  |
| **Undeclared** | - |  | 1.78 [1.50, 2.11] | <0.001 | 1.76 [1.49, 2.09] | <0.001 | 1.78 [1.50, 2.11] | <0.001 |
| **Alcohol misuse *** |  |  |  |  |  |  |  |  |
| **Yes** | 1.70 [1.50, 1.93] | <0.001 | - | - | 1.71 [1.51, 1.94] | <0.001 | 1.72 [1.52, 1.95] | <0.001 |
| **No** | 1 |  | - |  | 1 |  | 1 |  |
| **Benzodiazepine misuse*** |  |  |  |  |  |  |  |  |
| **Yes** | 1.43 [1.25, 1.63] | <0.001 | 1.45 [1.27, 1.65] | <0.001 | 1.44 [1.26, 1.64] | <0.001 | 1.44 [1.27, 1.64] | <0.001 |
| **No** | 1 |  | 1 |  | 1 |  | 1 |  |
| **Crack misuse*** |  |  |  |  |  |  |  |  |
| **Yes** | 0.96 [0.86, 1.07] | 0.44 | 0.98 [0.88, 1.09] | 0.74 | 0.98 [0.88, 1.09] | 0.68 | 0.98 [0.88, 1.09] | 0.70 |
| **No** | 1 |  | 1 |  | 1 |  | 1 |  |
| **Cocaine/amphetamines misuse*** |  |  |  |  |  |  |  |  |
| **Yes** | 1.09 [0.95, 1.26] | 0.22 | 1.11 [0.96, 1.28] | 0.16 | 1.10 [0.95, 1.27] | 0.18 | 1.11 [0.96, 1.28] | 0.17 |
| **No** | 1 |  | 1 |  | 1 |  | 1 |  |
| **Period following CJS referral** |  |  |  |  |  |  |  |  |
| **Yes** | 0.96 [0.84, 1.10] | 0.55 | 0.97 [0.85, 1.11] | 0.68 | - | - | 0.96 [0.84, 1.10] | 0.60 |
| **No** | 1 |  | 1 |  | - |  | 1 |  |

*Post declared problem use of (time dependent covariate)

**Appendix A continued: Covariates estimates for models in analysis**

| **Variable** | **Completion model** | | **Modality model** | | **Time dependent modality model** | |
| --- | --- | --- | --- | --- | --- | --- |
| **Gender** |  |  |  |  |  |  |
| **Male** | 1 |  | 1 |  | 1 |  |
| **Female** | 0.70 [0.62, 0.79] | <0.001 | 0.70 [0.62, 0.79] | <0.001 | 0.70 [0.62, 0.79] | <0.001 |
| **Age group** |  |  |  |  |  |  |
| **18-34** | 0.64 [0.57, 0.72] | <0.001 | 0.64 [0.57, 0.71] | <0.001 | 0.64 [0.57, 0.72] | <0.001 |
| **35-44** | 1 |  | 1 |  | 1 |  |
| **45-64** | 1.31 [1.14, 1.51] | <0.001 | 1.31 [1.13, 1.51] | <0.001 | 1.29 [1.12, 1.49] | <0.001 |
| **Injecting *** |  |  |  |  |  |  |
| **Yes** | 2.11 [1.89, 2.36] | <0.001 | 2.15 [1.92, 2.41] | <0.001 | 2.18 [1.95, 2.44] | <0.001 |
| **No** | 1 |  | 1 |  | 1 |  |
| **Undeclared** | 1.77 [1.49, 2.10] | <0.001 | 1.76 [1.49, 2.09] | <0.001 | 1.81 [1.52, 2.14] | <0.001 |
| **Alcohol misuse *** |  |  |  |  |  |  |
| **Yes** | 1.72 [1.52, 1.96] | <0.001 | 1.68 [1.48, 1.91] | <0.001 | 1.69 [1.49, 1.92] | <0.001 |
| **No** | 1 |  | 1 |  | 1 |  |
| **Benzodiazepine misuse*** |  |  |  |  |  |  |
| **Yes** | 1.44 [1.26, 1.64] | <0.001 | 1.45 [1.27, 1.65] | <0.001 | 1.46 [1.28, 1.66] | <0.001 |
| **No** | 1 |  | 1 |  | 1 |  |
| **Crack misuse*** |  |  |  |  |  |  |
| **Yes** | 0.98 [0.88, 1.09] | 0.68 | 0.96 [0.86, 1.06] | 0.41 | 0.96 [0.86, 1.07] | 0.43 |
| **No** | 1 |  | 1 |  | 1 |  |
| **Cocaine/amphetamines misuse*** |  |  |  |  |  |  |
| **Yes** | 1.11 [0.96, 1.27] | 0.17 | 1.09 [0.94, 1.25] | 0.26 | 1.09 [0.95, 1.26] | 0.22 |
| **No** | 1 |  | 1 |  | 1 |  |
| **Period following CJS referral** |  |  |  |  |  |  |
| **Yes** | 0.96 [0.84, 1.10] | 0.60 | 0.93 [0.81, 1.06] | 0.27 | 0.93 [0.81, 1.06] | 0.27 |
| **No** | 1 |  | 1 |  | 1 |  |

*Post declared problem use of (time dependent covariate)

**Appendix B Sensitivity analysis comparing periods in treatment with periods out after varying the treatment end date to two weeks later than the recorded date for those with an 'unplanned' discharge**

| **Variable** | **Pyears, thousand** | **Number of DRPs (%)** | **DRD rate, per 1,000 pyears** | **Unadjusted analysis** | | **Adjusted analysis** | |
| --- | --- | --- | --- | --- | --- | --- | --- |
|  |  |  |  | **Hazard ratio [95 % CI]** | **p-value** | **Hazard ratio [95 % CI]** | **p-value** |
| **Treatment status** |  |  |  |  |  |  |  |
| **In** | 310 | 918 | 3.0 [2.8, 3.2] | 1 | <0.001 | 1 | <0.001 |
| **Out** | 133 | 581 | 4.4 [4.0, 4.7] | 1.50 [1.35, 1.67] |  | 1.65 [1.48, 1.83] |  |

Adjusted for all variables in table 2 (main analysis)

**Appendix C Sensitivity analysis: Separate proportional hazard analysis for those in treatment 1st April 2005 (prevalent cohort) and those who entered treatment between 1st April 2005 to 31st March 2009 (incident cohort)**

**Prevalent cohort**

**Number of subjects =** **56,423; person years = 222,165**

|  |  |  |  | **Unadjusted analysis** | | **Adjusted analysis** | |
| --- | --- | --- | --- | --- | --- | --- | --- |
| **Variable** | **Pyears, thousand** | **Number of DRPs** | **DRP rate, per 1,000 pyears [95 % CI]** | **Hazard ratio [95 % CI]** | **p-value** | **Hazard ratio [95 % CI]** | **p-value** |
| **Treatment status** |  |  |  |  | |  | |
| **In** | 171 | 495 | 2.9 [2.6, 3.2] | 1 | <0.001 | 1 | <0.001 |
| **Out** | 51 | 256 | 5.0 [4.4, 5.7] | 1.74 [1.49, 2.03] |  | 2.10 [1.75, 2.52] |  |
| **Gender** |  |  |  |  |  |  |  |
| **Male** | 148 | 561 | 3.8 [3.5, 4.1] | 1 | <0.001 | 1 | <0.001 |
| **Female** | 75 | 190 | 2.5 [2.2, 2.9] | 0.67 [0.57, 0.79] |  | 0.76 [0.64, 0.89] |  |
| **Age group** |  |  |  |  |  |  |  |
| **18-34** | 109 | 259 | 2.4 [2.1, 2.7] | 0.59 [0.50, 0.69] | <0.001 | 0.58 [0.49, 0.68] | <0.001 |
| **35-44** | 80 | 322 | 4.0 [3.6, 4.5] | 1 |  | 1 |  |
| **45-64** | 34 | 170 | 5.1 [4.4, 5.9] | 1.26 [1.05, 1.52] |  | 1.36 [1.12, 1.64] |  |
| **Injecting *** |  |  |  |  |  |  |  |
| **Yes** | 87 | 386 | 4.4 [4.0, 4.9] | 1.89 [1.61, 2.21] | <0.001 | 1.84 [1.57, 2.16] | <0.001 |
| **No** | 113 | 102 | 4.6 [3.8, 5.6] | 1 |  | 1 |  |
| **Undeclared** | 22 | 102 | 4.6 [3.8, 5.6] | 1.98 [1.57, 2.48] |  | 1.85 [1.47, 2.33] |  |
| **Alcohol misuse *** |  |  |  |  |  |  |  |
| **Yes** | 25 | 135 | 5.4 [4.6, 6.4] | 1.74 [1.45, 2.10] | <0.001 | 1.65 [1.36, 1.99] | <0.001 |
| **No** | 197 | 616 | 3.1 [2.9, 3.4] | 1 |  | 1 |  |
| **Benzodiazepine misuse*** |  |  |  |  |  |  |  |
| **Yes** | 32 | 162 | 5.1 [4.4, 6.0] | 1.66 [1.40, 1.98] | <0.001 | 1.63 [1.37, 1.95] | <0.001 |
| **No** | 191 | 589 | 3.1 [2.9, 3.4] | 1 |  | 1 |  |
| **Crack misuse*** |  |  |  |  |  |  |  |
| **Yes** | 70 | 243 | 3.4 [3.0, 3.9] | 1.03 [0.88, 1.20] | 0.73 | 0.99 [0.85, 1.16] | <0.001 |
| **No** | 152 | 508 | 3.3 [3.1, 3.7] | 1 |  | 1 |  |
| **Cocaine/amphetamines misuse*** |  |  |  |  |  |  |  |
| **Yes** | 28 | 98 | 3.5 [2.9, 4.3] | 1.05 [0.85, 1.30] | 0.64 | 0.97 [0.78, 1.20] | <0.001 |
| **No** | 194 | 653 | 3.4 [3.1, 3.6] | 1 |  | 1 |  |
| **Period following CJS referral** |  |  |  |  |  |  |  |
| **Yes** | 28 | 106 | 3.8 [3.2, 4.6] | 0.94 [0.79, 1.11] | 0.19 | 1.10 [0.89, 1.35] | 0.40 |
| **No** | 194 | 645 | 3.3 [3.1, 3.6] | 1 |  | 1 |  |

*Post declared problem use of (time dependent covariate)

**Incident cohort**

**Number of subjects =114,740; person years= 222,165**

|  |  |  |  | **Unadjusted analysis** | | **Adjusted analysis** | |
| --- | --- | --- | --- | --- | --- | --- | --- |
| **Variable** | **Pyears, thousand** | **Number of DRPs** | **DRP rate, per 1,000 pyears [95 % CI]** | **Hazard ratio [95 % CI]** | **p-value** | **Hazard ratio [95 % CI]** | **p-value** |
| **Treatment status** |  |  |  |  | |  | |
| **In** | 136 | 395 | 2.9 [2.6, 3.2] | 1 | <0.001 | 1 | <0.001 |
| **Out** | 85 | 353 | 4.2 [3.8, 4.6] | 1.46 [1.26, 1.69] |  | 1.60 [1.38, 1.86] |  |
| **Gender** |  |  |  |  |  |  |  |
| **Male** | 156 | 601 | 3.9 [3.6, 4.2] | 1 | <0.001 | 1 | <0.001 |
| **Female** | 65 | 147 | 2.3 [1.9, 2.7] | 0.58 [0.49, 0.70] |  | 0.64 [0.53, 0.77] |  |
| **Age group** |  |  |  |  |  |  |  |
| **18-34** | 127 | 349 | 2.7 [2.5, 3.0] | 0.67 [0.57, 0.78] | <0.001 | 0.69 [0.59, 0.81] | <0.001 |
| **35-44** | 70 | 286 | 4.1 [3.6, 4.6] | 1 |  | 1 |  |
| **45-64** | 24 | 113 | 4.8 [4.0, 5.8] | 1.19 [0.95, 1.47] |  | 1.25 [1.00, 1.55] |  |
| **Injecting *** |  |  |  |  |  |  |  |
| **Yes** | 76 | 402 | 5.3 [4.8, 5.9] | 2.46 [2.11, 2.88] | <0.001 | 2.43 [2.07, 2.85] | <0.001 |
| **No** | 122 | 83 | 3.6 [2.9, 4.5] | 1 |  | 1 |  |
| **Undeclared** | 23 | 83 | 3.6 [2.9, 4.5] | 1.65 [1.29, 2.12] |  | 1.65 [1.29, 2.12] |  |
| **Alcohol misuse *** |  |  |  |  |  |  |  |
| **Yes** | 31 | 178 | 5.8 [5.0, 6.7] | 1.96 [1.65, 2.32] | <0.001 | 1.79 [1.51, 2.12] | <0.001 |
| **No** | 190 | 570 | 3.0 [2.8, 3.3] | 1 |  | 1 |  |
| **Benzodiazepine misuse*** |  |  |  |  |  |  |  |
| **Yes** | 28 | 126 | 4.5 [3.7, 5.3] | 1.39 [1.15, 1.68] | <0.001 | 1.26 [1.04, 1.53] | <0.001 |
| **No** | 193 | 622 | 3.2 [3.0, 3.5] | 1 |  | 1 |  |
| **Crack misuse*** |  |  |  |  |  |  |  |
| **Yes** | 92 | 316 | 3.4 [3.1, 3.8] | 1.03 [0.89, 1.19] | 0.005 | 0.97 [0.84, 1.13] | <0.001 |
| **No** | 129 | 432 | 3.4 [3.1, 3.7] | 1 |  | 1 |  |
| **Cocaine/amphetamines misuse*** |  |  |  |  |  |  |  |
| **Yes** | 30 | 129 | 4.3 [3.6, 5.1] | 1.32 [1.09, 1.59] | <0.001 | 1.23 [1.02, 1.49] | 0.02 |
| **No** | 190 | 619 | 3.3 [3.0, 3.5] | 1 |  | 1 |  |
| **Period following CJS referral** |  |  |  |  |  |  |  |
| **Yes** | 54 | 173 | 3.2 [2.8, 3.7] | 0.94 [0.79, 1.11] | <0.001 | 0.89 [0.75, 1.06] | 0.18 |
| **No** | 167 | 575 | 3.4 [3.2, 3.7] | 1 |  | 1 |  |

*Post declared problem use of (time dependent covariate)

**Appendix D Most common sequences of treatment interventions within a treatment-episode, first incident episodes only (number of subjects = 114,741; person years = 196,129)**

| **Intervention sequence** | **N (%)** | | **Person years, k (%)** | |
| --- | --- | --- | --- | --- |
| Pharmacotherapy-only | 68,714 | (60) | 82·0 | (68) |
| Psychosocial-only | 23,957 | (21) | 11·7 | (10) |
| Psychosocial followed by pharmacotherapy | 9,238 | (8) | 12·3 | (10) |
| Residential-only | 3,848 | (3) | 0·9 | (1) |
| Pharmacotherapy followed by psychosocial | 1,634 | (1) | 2·0 | (2) |
| Remainder of intervention sequences | 7,350 | (6) | 12·5 | (10) |

**Appendix E Post hoc analysis to assess the independent impact of CJS referrals from prison and the community**

| **Referral route** | **Treatment status** | **Person years (PY), 1,000** | **N of DRPs** | **DRP rate, per 1,000 PY** | **HR** | **aHR** |
| --- | --- | --- | --- | --- | --- | --- |
| **Referral route** | | | | | | |
| **Prison** | In | 7 | 35 | 4.7 [3.4, 6.6] | 1 | 1 |
|  | Out | 5 | 14 | 2.7 [1.6, 4.5] | 0.57 (0.30, 1.05) | 0.66 (0.35, 1.23) |
| **Community-CJS*** | In | 41 | 129 | 3.2 [2.7, 3.8] | 1 | 1 |
|  | Out | 28 | 101 | 3.6 [2.9, 4.3] | 1.13 (0.87, 1.47) | 1.28 (0.98, 1.67) |
| **Other referral** | In | 259 | 726 | 2.8 [2.6, 3.0] | 1 | 1 |
|  | Out | 102 | 494 | 4.8 [4.4, 5.3] | 1.76 (1.56, 1.97) | 1.91 (1.69, 2.14) |

*Arrest referral/DIP; DRR; probation
